# Effective treatment of anal cancer in the elderly with low-dose chemoradiotherapy

**DOI:** 10.1038/sj.bjc.6602486

**Published:** 2005-03-29

**Authors:** N Charnley, A Choudhury, P Chesser, R A Cooper, D Sebag-Montefiore

**Affiliations:** 1 Leeds Cancer Centre, Cookridge Hospital, Hospital Lane, Leeds, West Yorkshire LS16 6QB, UK

**Keywords:** anal cancer, chemoradiotherapy, low-dose radiotherapy, old age

## Abstract

Chemoradiotherapy (CRT) is accepted as the standard initial treatment for squamous cell anal cancer. However, frail elderly patients cannot always tolerate full-dose CRT. This paper reports the results of a modified regimen for this group of patients. In all, 16 patients with biopsy-proven squamous cell carcinoma of the anal canal or margin and performance status or co-morbidity precluding the use of full-dose CRT were included in this protocol. The median age was 81 (range 77–91). Patients received a dose of 30 Gy to the gross tumour volume plus 3 cm margin in all directions. Concurrent chemotherapy comprised 5-fluorouracil 600 mg m^−2^ given over 24 h on days 1–4 of radiotherapy. The treatment was well tolerated. All 16 patients completed treatment as planned. Only one patient experienced any grade 3 toxicity (skin). The local control at a median follow-up of 16 months was 73% (13 out of 16). The overall survival was 69% and disease-specific survival 86%. This is a well-tolerated regimen for elderly/poor performance patients with anal cancer, which can achieve high rates of local control and survival. Longer follow-up will determine whether these encouraging results are maintained.

Anal cancer is rare. Within the Yorkshire Cancer Network, with a population of 2.6 million, on average 30–40 patients with anal cancer are registered each year (Yorkshire Cancer Registry Report, 1996).

Chemoradiotherapy (CRT) is accepted as the standard initial treatment for squamous anal cancer with surgery used as salvage treatment for recurrent or persistent disease. Norman Nigro was the first to describe in a case report complete response in three patients treated with mitomycin C (MMC) and 5-fluorouracil (5-FU) combined with 30 Gy radiotherapy (Nigro *et al*, 1974). A subsequent report described the outcome for 45 patients treated with the same regimen with a complete response rate of 84% on biopsy 6 weeks after completion of treatment (Leichman *et al*, 1985).

A number of investigators reported encouraging results of initial nonsurgical treatment either using radiotherapy alone (Cummings *et al*, 1982; Eschwege *et al*, 1985; Papillon and Montbarbon, 1987; Martenson and Gunderson, 1993) or concurrent CRT (Flam *et al*, 1987; Cummings *et al*, 1991; John *et al*, 1996). This in turn led to three pivotal randomised control trials that investigated the role of CRT. The UKCCCR and the EORTC trials compared CRT with radiotherapy alone, whereas the RTOG trial compared CRT using 5-FU with and without the addition of MMC (Flam *et al*, 1996; UKCCCR, 1996; Bartelink *et al*, 1997). These results have led to CRT (using a total dose of radiation of at least 50 Gy combined with 5-FU and MMC) being established as the primary treatment of anal cancer.

In routine clinical practice, however, clinicians face the problem of deciding the best treatment option for elderly patients who are frail, suffer from significant medical co-morbid conditions or who are for other reasons considered unfit to undergo standard (full-dose) chemoradiation. In view of the impressive results obtained by Nigro and colleagues in 45 patients using a total dose of radiation of 30 Gy, we designed an institutional protocol taking into account the needs of this patient group and evaluated the toxicity and outcome using a total dose of 30 Gy of radiation delivered using an involved field approach with reduced dose concurrent 5-FU. The aim of this paper is to present the results of this protocol.

## PATIENTS AND METHODS

### Patient characteristics

Between January 2000 and December 2003, 101 patients with squamous cell carcinoma of the anal canal or margin were treated with CRT. In all, 15 of these patients were aged 75 or over but fit enough to be treated with standard dose CRT. However, 16 patients were deemed not suitable to be treated with our standard CRT protocol due to their age, performance status or co-morbidity. It is these patients who form the basis of this retrospective study. Six (37%) of the patients were males. All 16 patients had biopsy-proven squamous cell carcinoma situated in the anal canal (six patients), anal margin (six patients) or both (four patients). Patients were referred to a single consultant clinical oncologist (DSM) at Leeds Cancer Centre, Cookridge Hospital from the surrounding hospitals within the Yorkshire Cancer Network.

All patients were initially considered for standard dose CRT. However, in all patients there was a combination of factors, either significant co-morbidity (as outlined in Table 1), combined with their age, and/or performance status that in the opinion of the treating clinical oncologist meant the patient would not tolerate, or complete standard dose CRT without major treatment modification. Seven patients were of World Health Organisation (WHO) performance status (PS) 1, eight patients PS 2 and one patient PS 3. Patients were all treated according to an institutional protocol, as outlined below.

A pretreatment examination, including the anal margin, canal and inguino-femoral region was performed in all patients. Female patients routinely underwent vaginal examination as part of the initial assessment. The tumour site was defined as canal or margin dependent on the location of the majority of the disease. A full blood count and biochemical profile were also obtained in all patients. Of the 16 patients, 13 had metastatic disease excluded by CT scanning of the abdomen and pelvis and chest X-ray. Three patients did not undergo radiological assessment as they had tumours where the risk of metastatic disease was thought to be low, and in addition there were no symptoms or signs to suggest regional or metastatic spread. Patient and tumour characteristics are shown in Table 1. The median age was 81 years (range 77–91).

### Radiotherapy

Treatment was delivered as a single phase without a boost or planned gap. All patients were simulated and treated prone with a full bladder. Gross tumour volume (GTV) was defined as all visible macroscopic tumour and any nodal disease considered clinically or radiologically to be significant. In the perianal and inguinal area, this was marked with radio-opaque markers at the time of simulation. Moulded wax block bolus to the perianal skin was used during the treatment. Patients were planned using an orthogonal film or virtual simulation technique. Small bowel and rectal contrast were used routinely.

The radiotherapy fields were defined as the GTV plus a margin of 3 cm in all directions. For anal margin cancers with no extension into the anal canal, a direct field was used (five patients, median field dimensions 10 × 10). Seven patients without nodal disease were treated using either a three- or four-field planned technique (median field dimensions 17 × 14 × 15 cm^3^) and four patients were treated with parallel opposed fields (median field dimensions 14 × 16 cm^2^). Bolus was also applied to involved groin nodes. Photons were used for all patients. The dose prescription was 30 Gy in 15 fractions as an applied dose in the case of a direct field or the same dose prescribed to the ICRU intersection point for multiple field arrangements.

### Chemotherapy

Concurrent chemotherapy comprised 5-FU 600 mg m^−2^ in 1 litre N saline infused via a peripheral cannula over 24 h on days 1–4 of the first week of treatment. As part of the protocol, all patients were given prophylactic antibiotics (oral Ciprofloxacin 250 mg twice daily) at the commencement of treatment and continued until any areas of moist desquamation following CRT had healed. This is also standard policy in the current National Cancer Research Network ACT 2 phase III trial.

Patients were seen weekly during their radiotherapy to assess toxicity including a full blood count. Following completion of treatment, patients continued to be seen on a weekly basis until any toxicity had resolved.

### Follow-up

All patients were followed up jointly by the referring surgeon and oncologist. Patients were seen 3 monthly for the first 2 years and then 6 monthly until 5 years. At each visit a careful history and examination was performed, including assessment of the anal canal and margin and inguinal regions. Investigations (including radiological assessment) were only performed if the patient's symptoms or findings on examination suggested relapse.

### Statistical methods

Local failure was defined as recurrent or persistent disease more than 3 months following completion of CRT. Complete response was determined clinically and biopsy confirmation was not routinely obtained. Disease-free survival and overall survival were estimated using the method of Kaplan–Meier.

## RESULTS

### Treatment compliance and acute toxicity

All 16 patients completed CRT to the planned radiotherapy dose of 30 Gy in 15 fractions with 5-FU given concurrently in the first week. The calculated median GFR was 35 ml min^−1^ (range 18–54) (Cockcroft and Gault, 1976). Two patients were hospitalised throughout their treatment for social reasons. One patient required admission for symptom control part way through their radiotherapy. The remaining patients were all treated as outpatients apart from the initial 4 days of chemotherapy.

Although all patients experienced some degree of skin toxicity in the majority, this was mild erythema. Only one patient experienced grade 3 skin toxicity (Common Toxicity Criteria Version 2) and this patient required hospitalisation. One patient reported diarrhoea, which was mild and controlled by medication. No patients experienced haematological toxicity of any grade for the 4 weeks during treatment. One patient required a stoma prior to commencing treatment because of a recto-vaginal fistula. This has not been reversed. No patients have required stomas following completion of CRT. Hospital admission was not required in any patient for the management of post-treatment toxicity.

### Local control and survival

**Figure 1:**
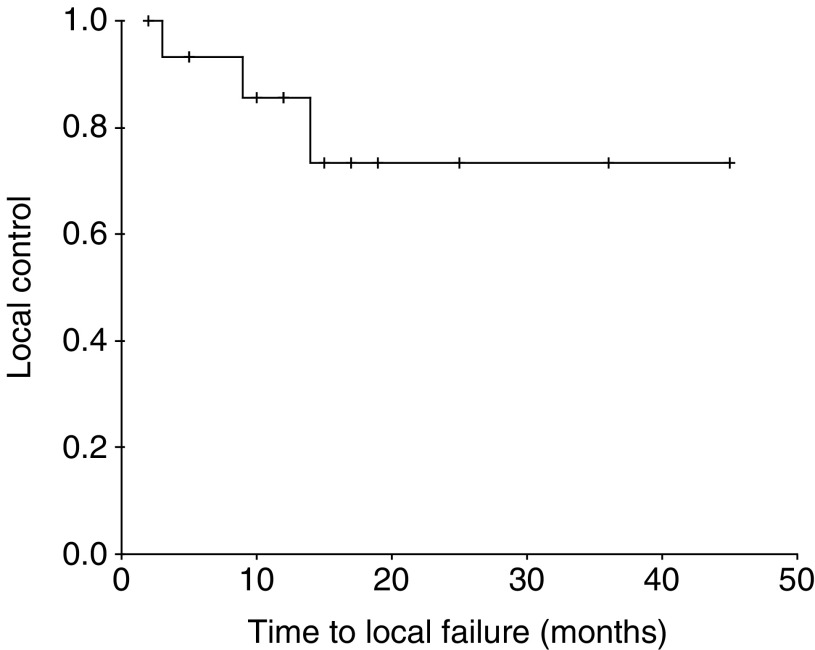
Local control.

The median follow-up is 16 months for all patients (range 2–45 months) and 17 months (range 5–45) for surviving patients. The disease status is given in Table 2. Three (19%) patients have failed locally, one with persistent disease and two with a local recurrence at 9 and 14 months. The actuarial local control rate is 73% (Figure 1). None of these patients were considered fit enough to undergo salvage surgery. Two of the patients who failed locally presented with locally advanced T3 tumours.

**Figure 2:**
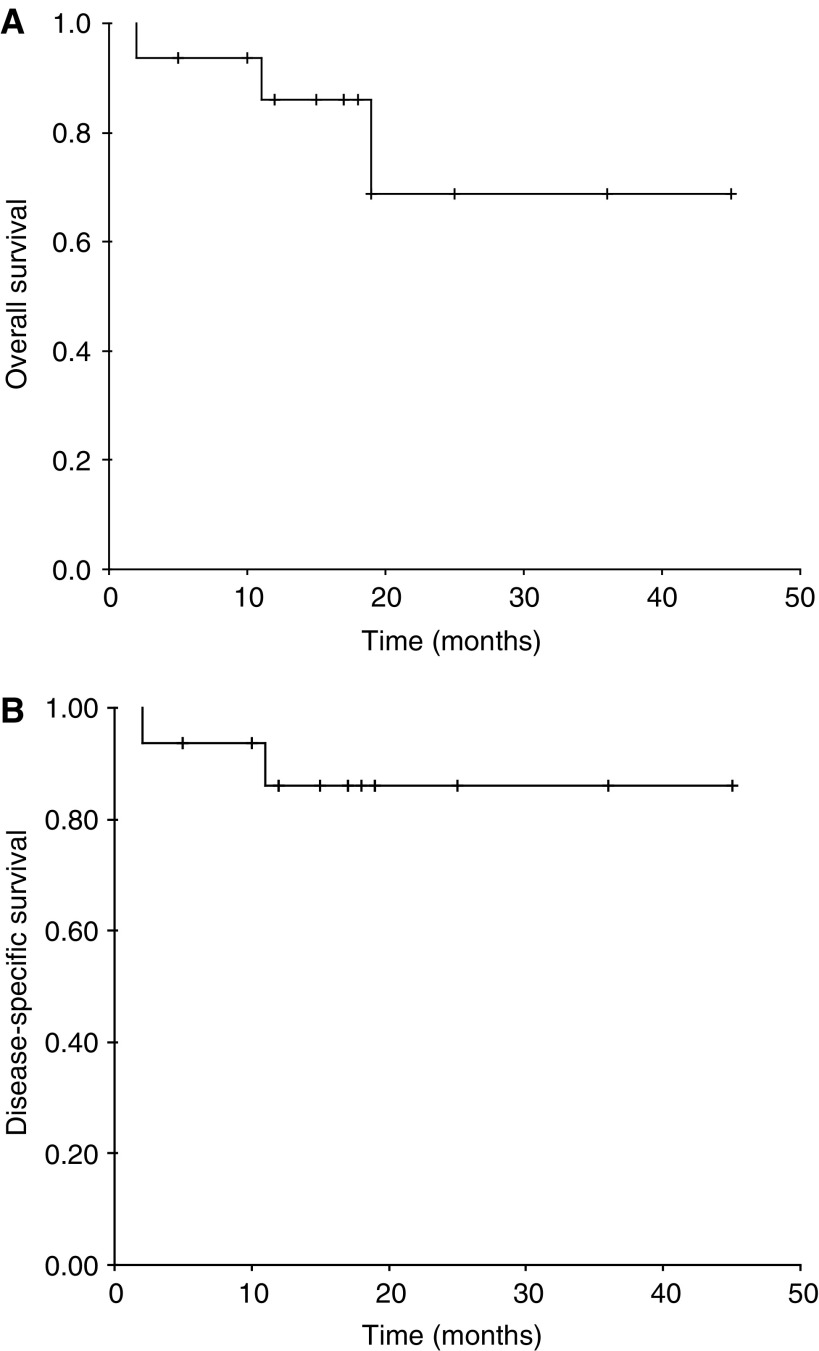
(**A**) Overall survival. (**B**) Disease-specific survival.

Three patients have died during follow-up, two from their anal cancer (one patient with local recurrence and the patient with persistent disease) and one from intercurrent illness (there was no evidence of recurrent tumour at the time of death). One patient is alive with local recurrence. The overall survival is 69% (Figure 2A) and the disease-specific survival 86% (Figure 2B).

## DISCUSSION

Chemoradiotherapy is the accepted primary treatment for squamous cell anal cancer, while abdominal perineal excision is reserved for persistent or confirmed locally recurrent disease. Two large randomised controlled trials have confirmed the superiority of CRT over radiotherapy alone in terms of both local control and colostomy-free survival (Flam *et al*, 1996; UKCCCR, 1996; Bartelink *et al*, 1997). A third trial demonstrated that a combination of 5-FU and MMC was shown to be superior to 5-FU alone when combined with radiation. In all of these studies the primary tumour and lymph nodes were treated with doses of 45 Gy with an additional boost dose of 5.4–25 Gy in selected patients. However, patients included in all three of these protocols were of a good PS and had a median age of approximately 60. The results reported in this paper are of a protocol specifically designed for a group of elderly and/or poorer PS patients with anal cancer who experience significant symptoms from their disease and can expect a reasonable survival but would not be able to tolerate full-dose CRT.

There have been several reports of the use of low-dose radiotherapy combined with chemotherapy to treat anal cancer. In Nigro's paper of 21 patients treated with 30 Gy in 15 fractions combined with MMC and 5-FU followed by surgery, 57% of patients had significant downstaging or no tumour at the time of surgery (Buroker *et al*, 1977). A subsequent report of 45 patients treated with the same regimen led to 84% having complete response and 89% remaining disease-free at a median follow-up of 50 months (Leichman *et al*, 1985). Smith *et al* treated 42 patients with the same regimen. In total, 90% of patients with T1 and T2 tumours were free of disease at 33 months, while only 38% of patients with T3 or T4 tumours achieved disease control (Smith *et al*, 1994). Low-dose radiochemotherapy has also been shown to be both effective and tolerable in HIV-infected patients with anal cancer (Peddada *et al*, 1997).

To date, there has been only one study which has specifically investigated the role of CRT in elderly patients with anal cancer. Valentini *et al* (1997) used CRT to treat a group of 17 patients with anorectal carcinoma who were all over the age of 75. Of note, patients with a poor PS or significant co-morbidity were excluded. Of these 17, seven patients had anal cancer and were treated with split course radiotherapy (24 Gy, 1.8 Gy per fraction repeated after a break of 4–5 weeks) combined with 5-FU and MMC in the first week of each treatment course. The treated volume included the tumour plus inguinal and pelvic nodes. Treatment was well tolerated, with only one patient developing grade 3 RTOG skin toxicity. Four of the seven patients with anal cancer achieved a complete response and the sphincter was preserved in six patients.

The protocol used in our patients was developed from the phase II studies reported by Nigros group and our own experience of using a total of 30 Gy to microscopic disease and a boost dose of 20 Gy to macroscopic disease without a planned gap in our standard regimen (Melcher and Sebag-Montefiore, 2003). To date we have not seen any isolated failures in the low-dose region. To our knowledge, our study is the first to report the results of a protocol of low-dose radiotherapy combined with chemotherapy specifically designed for use in elderly patients and/or poor performance patients. Although the results should be interpreted with some caution as the follow-up is still short, initial results suggest that this modified regimen can not only achieve good symptom control but also has a high chance of local control. Longer follow-up of this group of elderly patients will determine whether the local control of 73%, similar to that seen in our patients treated with high-dose radiotherapy, is maintained.

Our protocol was well tolerated by this group of elderly frail patients. All patients completed their CRT as planned with minimum toxicity and only one grade 3 skin toxicity. No patients required admission for haematological or gastrointestinal toxicity. As expected, the greatest treatment-related morbidity was skin toxicity, but in all patients this was easily managed with appropriate skin care and simple analgesics.

Of the patients treated, three have developed local recurrence. Of these three, two had large T3 tumours at presentation. Other groups have reported similar findings of reduced local control for patients with more advanced tumours (Cummings *et al*, 1991; Melcher and Sebag-Montefiore, 2003). For these patients there are a number of possible ways to improve our regimen without increasing the dose of radiotherapy. The dose of 5-FU could be increased, although this should be done with caution. A second method of intensifying the regimen would be to add MMC if renal function is adequate. Of note, only a small proportion of our patients would have had adequate enough renal function to receive MMC. Furthermore, a significant risk of haematological toxicity can be anticipated in patients receiving 5-FU and MMC compared to patients receiving 5-FU alone (Flam *et al*, 1996). Finally, by substituting the 5-FU for an oral fluoropyrimidine, the regimen could be made totally out-patient based and therefore more convenient for patients and radiotherapy departments. This approach is under consideration for fluoropyrimidine-based CRT in rectal cancer and would equally apply to anal cancer (Crane and Sargeant, 2004).

## CONCLUSION

With an aging population, the number of cases of anal cancer in elderly frail patients is likely to increase. Many of these patients are not able to tolerate full-dose CRT, but are expected to survive long enough to require treatment. Treatment with single or short hypofractionated regimens might undertreat these patients, resulting in patients dying with painful, uncontrolled local disease. The availability of a low-toxicity, relatively uncomplicated regimen means that more patients can be offered treatment, which not only improves symptoms but also offers the prospect of long-term tumour control.

## Figures and Tables

**Table 1:** Pretreatment characteristics, radiotherapy details

	**Age**	**Stage**	**Pre-morbidity**	**Radiotherapy details**
1	77	T2N0	Previous hysterectomy Hypertension	P
2	78	T3N0	Severe learning difficulties	S
3	91	T2N0	Aortic valve diseaseHeart failure	S
4	83	T2N0	Recent fracture of neck of femur	P
5	82	T2N0	IHD	S
6	78	T2N0	NIDDMHypertension	P
7	85	T4N2	Previous breast cancer	PO
8	88	T3N3	Performance status 3	PO
9	78	T3N0	IHD	P
10	80	T2N0	Metastatic prostate cancer	P
11	79	T2N0	IHD	PO
12	82	T2N0	IHDVascular disease	S
13	81	T2N0	NIDDMHypertensionVascular disease	P
14	83	T3N0	Emphysema	P
15	89	T2N0	HypertensionAsthma	PO
16	81	T3N0	Previous CVA	S

P=planned three-field technique; S=single direct field; PO=parallel opposed technique; IHD=ischaemic heart disease; NIDDM=non-insulin-dependent diabetes; CVA=cardiovascular accident.

**Table 2:** Patient outcome

**Patient**	**Local disease status**	**Status**
1	ND (15 months)	A (15 months)
2	ND (10 months)	A (10 months)
3	LR (9 months)	A (17 months)
4	ND (12 months)	A (12 months)
5	ND (16 months)	A (16 months)
6	ND (22 months)	A (22 months)
7	ND (5 months)	A (5 months)
8	ND (13 months)	A (13 months)
9	ND (18 months)	A (18 months)
10	ND (5 months)	A (5 months)
11	ND (36 months)	A (36 months)
12	ND (37 months)	A (37 months)
13	ND (1 month)	D (2 months)
14	LR (14 months)	D (19 months)
15	ND (45 Months)	A (45 months)
16	PD (3 months)	D (11 months)

ND=no disease; LR=local relapse; PD=persistent disease; A=alive; D=dead.

Times in brackets are the times of recurrence or death or times of last follow-up.
